# Contribution of NKT cells to the immune response and pathogenesis triggered by respiratory viruses

**DOI:** 10.1080/21505594.2020.1770492

**Published:** 2020-05-28

**Authors:** Emma Rey-Jurado, Karen Bohmwald, Nicolás M.S. Gálvez, Daniela Becerra, Steven A. Porcelli, Leandro J. Carreño, Alexis M. Kalergis

**Affiliations:** a Millennium Institute on Immunology and Immunotherapy, Departamento de Genética Molecular y Microbiología, Facultad de Ciencias Biológicas, Pontificia Universidad Católica de Chile, Santiago, Chile; b Department of Microbiology and Immunology, and Department of Medicine, Albert Einstein College of Medicine, Bronx, NY, USA; c Millennium Institute on Immunology and Immunotherapy, Programa de Inmunología, Instituto de Ciencias Biomédicas, Facultad de Medicina, Universidad de Chile, Santiago, Chile; d Departamento de Endocrinología, Facultad de Medicina, Pontificia Universidad Católica de Chile, Santiago, Chile

**Keywords:** Human respiratory syncytial virus, human metapneumovirus, pulmonary inflammation, viral infection, natural killer T cells

## Abstract

Human respiratory syncytial virus (hRSV) and human metapneumovirus (hMPV) cause acute respiratory tract infections in children worldwide. Natural killer T (NKT) cells are unconventional T lymphocytes, and their TCRs recognize glycolipids bound to the MHC-I-like molecule, CD1d. These cells modulate the inflammatory response in viral infections. Here, we evaluated the contribution of NKT cells in both hRSV and hMPV infections. A significant decrease in the number of neutrophils, eosinophils, and CD103^+^DCs infiltrating to the lungs, as well as an increased production of IFN-γ, were observed upon hRSV-infection in CD1d-deficient BALB/c mice, as compared to wild-type control mice. However, this effect was not observed in the CD1d-deficient BALB/c group, upon infection with hMPV. Importantly, reduced expression of CD1d in CD11b^+^ DCs and epithelial cells was found in hRSV -but not hMPV-infected mice. Besides, a reduction in the expression of CD1d in alveolar macrophages of lungs from hRSV- and hMPV-infected mice was found. Such reduction of CD1d expression interfered with NKT cells activation, and consequently IL-2 secretion, as characterized by *in vitro* experiments for both hRSV and hMPV infections. Furthermore, increased numbers of NKT cells recruited to the lungs in response to hRSV- but not hMPV-infection was detected, resulting in a reduction in the expression of IFN-γ and IL-2 by these cells. In conclusion, both hRSV and hMPV might be differently impairing NKT cells function and contributing to the immune response triggered by these viruses.

## Introduction

Acute respiratory tract infections constitute a major cause of morbidity and mortality worldwide for children under 5 years old, regardless of their economic status and with similar incidence rates in both industrialized and developing countries [1].Human respiratory syncytial virus (hRSV), recently renamed human orthopneumovirus [2], and human metapneumovirus (hMPV), are two of the most relevant microbiological agents causing ARTIs in children worldwide [1]. Both viruses are responsible for the highest rates of hospitalizations due to bronchitis and pneumonia[3]. In addition, clinical manifestations and diagnosis in children are similar for both hRSV- and hMPV-infections [4].

Both hRSV and hMPV induce a Th_2_-like skewed inflammatory T cell response, characterized by the secretion of high levels of IL-4 and IL-13 [5,6]. Such cytokine pattern promotes the recruitment of infiltrating inflammatory cells to the lungs, among which are included neutrophils, eosinophils, and monocytes; both in humans and -similarly- in mice [7]. Massive inflammatory cell infiltration can obstruct the airways and cause remarkable lung damage [8]. Such unbalanced cytokine production leads to a poor viral clearance. Further, it has been reported that hRSV infection does not elicit a memory CD8^+^T cell response in certain humans [9,10], rendering individuals susceptible to reinfections. Conversely, hRSV infection induces CD8^+^ T cell response and may mediate immunopathology in mice[11]. Interestingly, both hRSV and hMPV impair the capacity of dendritic cells (DCs) to expand and activate naïve T cells [12–16]. Particularly for hRSV, it is thought that the mechanism of inhibition of T cell activation relays on the impairment of a proper assembly of an effective immunological synapse between DCs and T-cells [13,14]. It has been reported that hMPV induces higher levels of neutrophils infiltration, activated NK cells and a more severe clinical illness than hRSV in BALB/c infected mice [17]. In summary, both hRSV and hMPV elicit detrimental T cell responses. Importantly, B cells and Natural Killer T (NKT) cells mediate such effector T cell response in hRSV and hMPV T cell priming.

NKT cells are a non-conventional subset of T cells that exhibit characteristics of both conventional T cells, such as a T cell receptor (TCR), and Natural Killer (NK) cells, such as NK receptors CD161 (NK1.1), NKG2D, and Ly-49A^18^. CD1d-restricted NKT cells are classified into two main subsets Type I and Type II [18,19]. The most studied subpopulation, known as invariant NKT (iNKT) -the Type I NKT cells-, express an invariant TCR that recognizes lipids and glycolipids bound to the MHC-I-like molecule CD1d [18,20]. Type II NKT cells express a broader TCR repertoire. CD1d is expressed by a great number of cells including DCs, B cells, macrophages, and some epithelial cells [21]. Since CD1d is required for the development and selection of NKT cells, CD1d-deficient mice models lack both type I and type II NKT cells. Several viruses, such as herpes simplex virus-1 (HSV-1) and human immunodeficiency virus (HIV) have been reported to interfere with CD1d expression, thus modulating the innate/inflammatory immune response promoted by this molecule [22]. Activation of NKT cells is mainly mediated by CD1d ligation, though CD1d-independent mechanisms have also been described, mainly associated with a cytokine-dependent activation [23]. CD1d-dependent activation of NKT cells requires antigen presentation –such as lipids loaded onto the CD1d molecule-, and in a lesser extent, IL-12 production and CD40/CD40L interactions [23]. As stated above, it has been described that cytokines secreted by antigen-presenting cells (APC), such as DCs, in response to viral infection, are enough to induce the lipid-independent NKT activation [23]. Following NKT activation, these cells rapidly secrete a broad range of cytokines including those typically related with Th_1_ (IFN-γ and TNF-α) and Th_2_ T cell responses (IL-4, IL-5, and IL-13) [24].

The most characterized NKT cell ligand is the marine sponge-derived α-galactosylceramide (α-GalCer) [25]. It has been found that α-GalCer modulates the immune response during infections by *Streptococcus pneumonie* [25] and H1N1 influenza virus, among others [26]. Limited research regarding the role of these NKT cells during hRSV infection has been performed, showing that these cells may be essential to modulate the infection with this virus [27,28]. Further, although hMPV shares several similarities in the associated-disease and the immune response with hRSV, the contribution of NKT cells during the infection with this virus remains unknown. Therefore, here we evaluated the contribution of CD1d and the contribution of NKT cells during both hRSV and hMPV infections.

Here we show that reduced inflammatory cell infiltration and lung damage was observed in CD1d-deficient BALB/c (CD1d^-/-^) mice infected with hRSV, as compared to wild-type control animals. These phenomena were not observed in hMPV-infected mice. Furthermore, we detected a reduction in the surface expression of CD1d in CD11b^+^ DCs, epithelial cells, and alveolar macrophages in hRSV-infected mice, while this reduction of CD1d was only seen in alveolar macrophages during an infection of mice with hMPV. Besides, when we co-cultured either hRSV- or hMPV-infected DCs with iNKT cells hybridomas, an impairment in the capacity of iNKT cells to secrete IL-2 was observed; suggesting that both viruses hinder iNKT cell function.

## Material and methods

### Ethics statement

All mice experiments were conducted in agreement with ethical standards and according to the local animal protection law number 20.800. All experimental protocols were according to the Sanitary Code of Terrestrial Animals of the World Organization for Animal Health (OIE, 24^th^ Edition, 2015), were reviewed, and approved by the Scientific Ethical Committee for Animal and Environment Care of the *Pontificia Universidad Católica de Chile* (Protocol number 160404004).

### Mice

Original colonies of wild-type (WT) BALB/c, CD1d-deficient BALB/c (CD1d^-/-^), and wild-type C57BL/6 mice were initially obtained from the Jackson Laboratory (Bar Harbor, ME). Animals were subsequently bred and maintained at the pathogen-free animal facility of the *Pontificia Universidad Católica de Chile* (Santiago, Chile). A total of 20 WT BALB/c, 18 CD1d^-/-^ and 6 WT C57BL/6 mice were used for all the experiments. Male mice used during *in vivo* experiments were 6- to 8-weeks-old for all the strain used.

### Viruses

HEp-2 and LLC-MK2 cell monolayers were grown in T75 flasks with DMEM medium (10% FBS) or OPTIMEM medium (5% FBS), respectively. At 70–80% confluence, T75 flask of HEp-2 cells was inoculated with hRSV MOI = 1 (strain 13018–8, a clinical isolate provided by the Public Health Institute of Chile) in DMEM (1% FBS). Similarly, LLC-MK2 cells were inoculated with hMPV MOI = 1 (strain CZ0107, a clinical isolate provided by the Public Health Institute of Chile) in OPTIMEM supplemented with CaCl_2_ (100 mg/ml). After 2 h medium was replaced, and cells were finally cultured for 72 hours at 37°C until the cytopathic effect was observed. During virus collection, cells were scraped, and the infectious media were pooled and centrifuged at 500 g for 5 min to remove cell debris. In parallel, supernatants of non-infected monolayers cells were collected as previously described and used as noninfectious control (mock) for each respective virus.

### hRSV and hMPV challenge in vivo

Twenty 6- to 8-week-old male wild-type and 18 CD1d^-/-^ mice were anesthetized intraperitoneally (i.p.) with a single dose of ketamine/xylazine (20 mg/kg and 1 mg/kg, respectively) and challenged intranasally (i.n.) with 1 × 10^6^ PFU of hRSV (13018-8 strain) or 1 × 10^6^ PFU of hMPV (strain CZ0107) or the respective mock (as noninfectious control) in a final volume of 100 µL. Animal body weight and clinical scores were recorded up until 3-days post-infection (p.i.), when animals were euthanized. Such endpoint was selected based on previous data from our laboratory, showing higher NKT cells on day 3 p.i. as compared to day 6 p.i.

### Flow cytometry analysis

At day 3 p.i., mice were terminally anesthetized by intraperitoneal (i.p.) injection of a mixture of ketamine and xylazine (100 mg/kg and 5 mg/kg, respectively). Animals were euthanized at day 3, since hRSV- and hMPV-associated disease were previously described at that time point [29,30]. The left lung was occluded using a kelly hemostatic forceps, and bronchoalveolar lavage fluids (BALF) were obtained by gently instilling intratracheally 1 mL of 5% FBS-PBS three times, as previously described [31]. Next, lung samples were collected. For this, after BALF collection, the right lung was cut into small pieces and incubated for 1 h at 37°C in PBS containing 1 mg/mL of collagenase type IV (Thermo Fisher Scientific, Catalog No. 17104–019). To analyze the infiltration of inflammatory cells into the lung interstitium, lung cells were processed for flow cytometry staining and stained using the following antibodies: anti-Ly-6G FITC (clone 1A8), anti-Siglec-F PE (clone ESO-2440), anti-Ly-6C PerCPCy5.5 (clone HK1.4), anti-CD11c APC (clone HL3), anti-CD11b PECy7 (clone M1/70), anti-I/A-I/E (MHC-II) APCCy7 (clone M5/114.15.2, BioLegend), anti-CD8α FITC and APCCy7 (clone 53–6.7), anti-TCRβ APC (clone H57-597), anti-CD4 PECy7 (clone RM4-5), anti-CD45 FITC (clone 3O-F11), anti-B220 PE (clone RA3-6B2), anti-CD1d Bv395 (clone 1B1). Absolute cell counts were determined using CountBright^TM^ absolute counting beads in a 1:30 dilution in a maximum volume of 100 μl and acquired in a LSR FORTESSA X-20 flow cytometer. Data were analyzed using FlowJo v X 10.0.7 (FlowJo, LLC). CD103^+^ DCs has been previously reported to be CD11b and CD64 negative cells and positive for CD24 [32]. Also, additionally to these surface markers, CD103^+^ DCs population can be differentiated from plasmacytoid DCs with CD11c^high^ MHC-II^high^ surface markers [32]. The gating strategy is shown in the Supplementary Figure 2.

To analyze the expression of CD1d in monocytes, alveolar macrophages, CD103^+^ DCs, and CD11b DCs^+^ in the lungs, cells were stained using the following antibodies: anti-CD11b FITC (clone M1/70), anti-CD11c PeCy7 (clone HL3), anti-CD1d BV395 (clone 1B1), anti-Ly6G PE (clone 1A8), anti-MHC-II Bv605 (clone M5/114.15.2), anti-CD24 Bv711 (clone M1/69), anti-CD64 Alexa 647 (clone X54-5/7.1) and anti-CD45 BV786 (clone 30-F11). To analyze the expression of CD1d in epithelial cells, the following antibodies were used: Anti-CD45 FITC (clone 30-F11), anti-Epcam PE (clone G8.8) and anti-CD1d BV395 (clone 1B1). Cells were acquired using BD LSR FORTESSA X-20 flow cytometer. The gating strategy used to select epithelial cells, undifferentiated monocytes, alveolar macrophages, CD103^+^ DCs, and CD11b^+^ DCs was used as previously described [32] (Supplementary Figure 3). Positivity for CD1d marker was assessed using negative control of staining. Then, CD1d expression was evaluated with the mean fluorescence intensity (MFI) of the positive CD1d selection (Supplementary Figure 3). Data were analyzed using FlowJo v X 10.0.7 (FlowJo, LLC). For intracellular staining of iNKT cells from lungs, cells suspensions were incubated with a cocktail of PMA, Ionomycin, BFA, and Monensin for 5 hours. Both monensin and BFA were used together to prevent protein secretion of all the cytokines studied. Both PMA and ionomycin were used as unspecific stimulation to obtain the lung-resident NKT-cytokine expression in the infected-mice. Unstimulated cells with BFA and Monensin were included as controls in these experiments. After incubation, plates were stored at 4ºC O.N. Twelve hours later, cells were washed and stained with BD Horizon Fixable Viability Stain 700 staining and surface T cell markers (anti-CD45 BV510 (clone 3O-F11); anti-T cell receptor βFITC (clone H57-597); α-GalCer*–*loaded CD1d tetramer APC. Then, after 2 h of incubation cells were fixed using Cytofix-Cytoperm (BD, Cat 554723) for 10 min, permeabilized using permeabilization buffer (BD, Cat 51–2090KC) and stained for 2 h with anti-IFN-γ PE (clone XMG1.2), anti-IL-2 APCCy7 (clone JE6-51-14), and anti-IL-4 PeCy7 (clone BVD6-24G2). After three washes with the same permeabilization buffer, cells were resuspended in PBS and immediately acquired using a BD LSR FORTESSA X-20 flow cytometer. To analyze the percentage of cytokines expressed in NKT cells, we adjust the gate according to the fluorescence minus one (FMO) control. The gating strategy is shown in Supplementary Figure 4.

### Real-time PCR for viral RNA detection

To determine the viral loads of infected mice, we isolated total RNA from a small piece of the left lung using TRIzol reagent®, according to the manufacturer’s instructions. Complementary DNAs (cDNA) was synthesized from 1 µg of total RNA using the iScript (Biorad) Reverse Transcription System kit and random primers. Quantitative Real-time PCR reactions were carried out with 500 ng of cDNA using Fast SYBER Green Master Mix, according to the manufacturer’s instructions. Primers used for N-hMPV gene detection in q-RT-PCR reactions were 5ʹ-ACAGCAGATTCTAAGAAACTCAGG-3ʹ (forward), and 5ʹ-TCTTTGTCTATCTCTTCCACCC-3ʹ (reverse) and primers used for N-hRSV gene detection were 5′-GAGACAGCATTGACACTCCT-3′ (forward) and 5′-CGATGTGTT GTTACATCCACT-3′ (reverse). Detection of mouse β-actin was used as a housekeeping reference gene using primers 5ʹ-AGGCATCCTGACCCTGAAGTAC-3ʹ (forward) and 5ʹ-TCTTCATGAGGTAGTCTGTCAG-3ʹ (reverse) as previously described [8]. Standard curves for absolute quantification were generated from increasing concentrations of N-hMPV, N-hRSV, and β-actin templates. Finally, the quantification of viral load was expressed as N-hMPV or N-hRSV transcript copies per 5,000 β-actin transcript copies.

### Lung histopathology

In order to perform histopathology analyses without losing significant tissue architecture, before BALF collection the major left bronchus of the left lung was clamped using a 10 cm Kelly hemostatic forceps. After obtaining the BALF of the right lung, the left lung was fixed with 4% paraformaldehyde (PFA) and then paraffined embedded using a Leica ASP300 S enclosed, automatic tissue processor (Leica Microsystems, Wetzlar, Germany). Then, 5 μm-thick tissue sections were obtained using a Microm HM 325 Rotary Microtome (Thermo Scientific), mounted and stained for histopathology analyses using hematoxylin & eosin (H&E). Histopathology score of lung tissue was performed using parameters of the cell infiltrate, hemorrhage, edema, and lung damage previously described [33].

### iNKT cell activation assay

For the iNKT cell activation assay, WT C57/BL6 mice were used since the available iNKT hybridoma had a C57/BL6 mice background and because are widely used for the antigen presentation assays [14,34–36]. Bone marrow-derived DCs from WT C57BL/6 mice were prepared as described previously [37]. Briefly, DCs were grown in RPMI 1640 medium with 10% FBS, supplemented with murine GM-CSF. On day 5 of culture, DCs were inoculated either with mock, hRSV or hMPV at MOI = 5 for 2 h. After that, the cells were washed and pulsed with α-GalCer at different concentrations (1 μM, 100 nM, 10 nM, and 1 nM) for 24 h or treated with vehicle (VH; 1 X PBS 0.005% DMSO). DCs were then co-cultured with DN3A4-1.2 iNKT cells hybridomas [38] at a DCs:iNKT cell ratio equal to 1:2 for 24 h. Secretion of IL-2, IL-12, IL-4, and IFN-γ was measured after 24 h of DC-NKT hybridoma co-culture by ELISA (BD OptEIA™).

### Cytokine ELISA

Using ELISA (BD OptEIA™) techniques, IFN-γ, IL-5, IL-6, IL-1β, and IL-10 were detected in the supernatant of BALF samples.

### Statistical analyses

All statistical analyses were performed using GraphPad Prism 5.0 (GraphPad Sofware). Statistical significances were assessed using Kruskal–Wallis and Mann–Whitney *U* non-parametric tests with a posteriori Bonferroni test. Differences were considered significant when p-value< 0.05.

## Results

### The CD1d expression is modulated in alveolar macrophages, epithelial cells, and infiltrating CD11b^+^ DCs during respiratory virus infections

**Figure 1.**
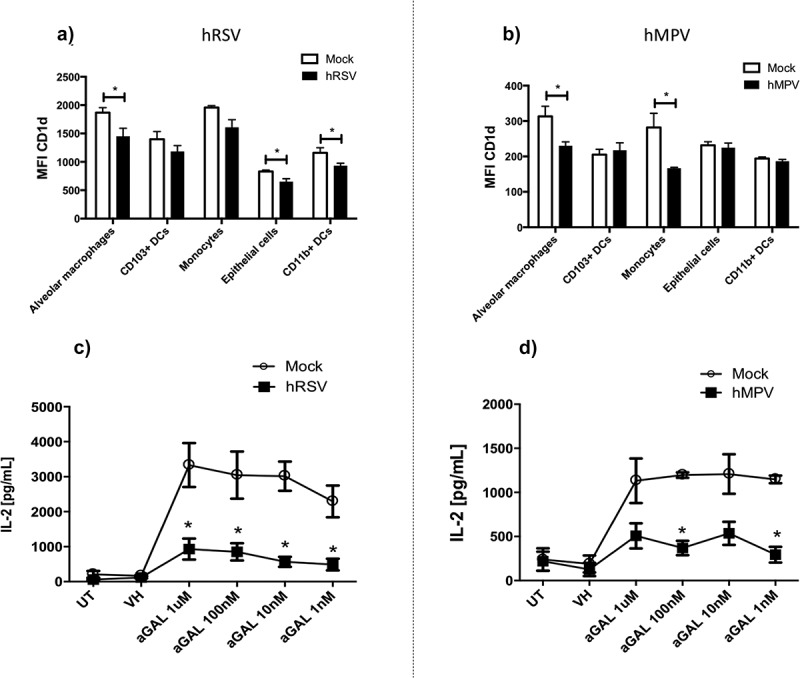
Expression levels of CD1d in infiltrated cells into the lungs from hRSV- and hMPV-infected mice and IL-2 secretion by NKT cells hybridoma *in vitro*. Wild-type [WT] BALB/c mice were infected with 1 × 10^6^ PFU of hRSV (a) or hMPV (b) and 3-days post-infection lungs were removed, homogenized and cells were stained to detect the expression of CD1d in alveolar macrophages (CD45^+^CD11b^−^CD11c^+^CD64^+^CD24), CD103^+^ DCs (CD45^+^CD11b^−^CD11c^+^CD64^−^CD24^+^), monocytes (CD45^+^CD11b^+^MHCII^−^CD64^−/low^), epithelial cells (CD45^−^Epcam^+^), and DCs CD11b^+^ (CD45^+^CD11b^+^CD11c^+^MHCII^+^CD24^−^CD64^−^). The mean fluorescence intensity (MFI) for CD1d on these cells was analyzed by flow cytometry. Combined data from two independent experiments (n = 4 to 5 mice per group and independent experiment) are shown. Kruskal–Wallis test and Mann–Whitney *U* tests were performed to assess statistical differences. * P < 0.05 ** P < 0.01 *** P < 0.001. Bars represent mean ± SEM. Pre-pulsed α-GalCer and hRSV- (c) or hMPV- (d) infected DCs from wild-type C57BL/6 mice were co-cultured with iNKT cells hybridoma and IL-2 production was evaluated by ELISA. UT: Untreated; VH: Vehicle; α-GAL: α-Galcer. Combined data from three independent experiments (n = 2–3 treatment per group and experiment) are shown. Kruskal–Wallis and Mann–Whitney *U* test were performed to assess statistical differences. * P < 0.05. Lines represent mean ± SEM.

Since it has been previously reported that the expression of CD1d in specific cells could promote a Th_1_-like response in the NKT cells population, we evaluated whether the expression of this molecule was modulated in cells infiltrating the lungs of hRSV- and hMPV-infected mice (Figure 1). Wild-type BALB/c mice were infected with hRSV or hMPV or inoculated with mock, and the disease progression was monitored for 3 days. Importantly, we detected a significant decrease of CD1d expression in alveolar macrophages, CD11b^+^ DCs, and epithelial cells in hRSV-infected mice as compared to mock-treated mice (Figure 1(a)). In contrast, CD1d expression in monocytes and CD103^+^ DCs in lungs from hRSV-infected mice was similar to the mock-treated mice (Figure 1(a)). Additionally, the expression of CD1d was reduced in alveolar macrophages and monocytes from hMPV-infected mice as compared to mock-treated mice (Figure 1(b)). Importantly, no statistical differences were found for the expression of CD1d in all the other cell types evaluated in hMPV-infected as compared to mock-treated mice (Figure 1(b)). This first piece of data suggests that iNKT cell play a role during the pathogenesis of both viruses, as their infection modulates the expression of the CD1d molecule during the disease.

### In vitro *capacity of infected DCs to promote IL-2 mediated-iNKT cells activation is impaired by hRSV and hMPV*

To evaluate whether hRSV and hMPV might be able to impair iNKT cells activation, we performed *in vitro* NKT cell activation assays. DCs pulsed with α-GalCer and then infected with either hRSV or hMPV (obtained from the bone marrow of C57BL6 mice), were co-cultured with iNKT cell hybridomas. No difference in CD1d expression in DCs was found between the experimental groups. Upon 24 h of co-culture the secretion of IL-2, IFN-γ, IL-12, and IL-4 was quantified by ELISA assays (Figure 1(c, d), Supplementary Figure 1C-F). Significant levels of IL-2 secretion were observed in iNKT cells co-cultured with α-GalCer pre-pulsed and mock-treated DCs, in an α-GalCer dose-dependent manner only for hRSV infection. In this line, the highest concentration of IL-2 detected was obtained in the 1µM α-GalCer treatment, as compared to all the other lipid concentrations (Figure 1(c,d)). This reduction in the secretion of IL-2 suggest that α-GalCer-loaded DCs that were infected with either hRSV or hMPV may exhibit a reduced capacity to induce the activation of iNKT cells; as secretion of IL-2 is an immediate response upon NKT cells activation (Figure 1(c,d)). This phenomenon is not associated to the capacity of DCs to secrete IL-12 -and therefore induce the secretion of IL-2 by iNKT cells-, as DCs infected with both hRSV and hMPV does not exhibit differences in their IL-12 secretion profile, as compared to mock-treated DCs (Supplementary Figure 1A and 1B). NKT cells hybridoma co-cultured with α-GalCer-loaded DCs that were infected either with hRSV or hMPV show a tendency to secrete lower amounts of IFN-γ, as compared to mock control groups (Supplementary Figure 1 C and 1D). Regarding to the IL-4 secretion by NKT cells hybridoma, we did not detect differences between α-GalCer-loaded DCs infected with hRSV and the mock control groups. However, we detected that α-GalCer-loaded DCs infected with hMPV tended to secrete more IL-4 than the mock control groups (Supplementary Figure 1E and 1F). Considering all this and in relation with the down-modulation of CD1d detected previously, both hRSV- and hMPV-infections seem to impair the production of IL-2 by iNKT cells in response to α-GalCer-loaded APC.

### Absence of CD1d -and accordingly NKT cells- is beneficial against hRSV- but not hMPV-infection

**Figure 2.**
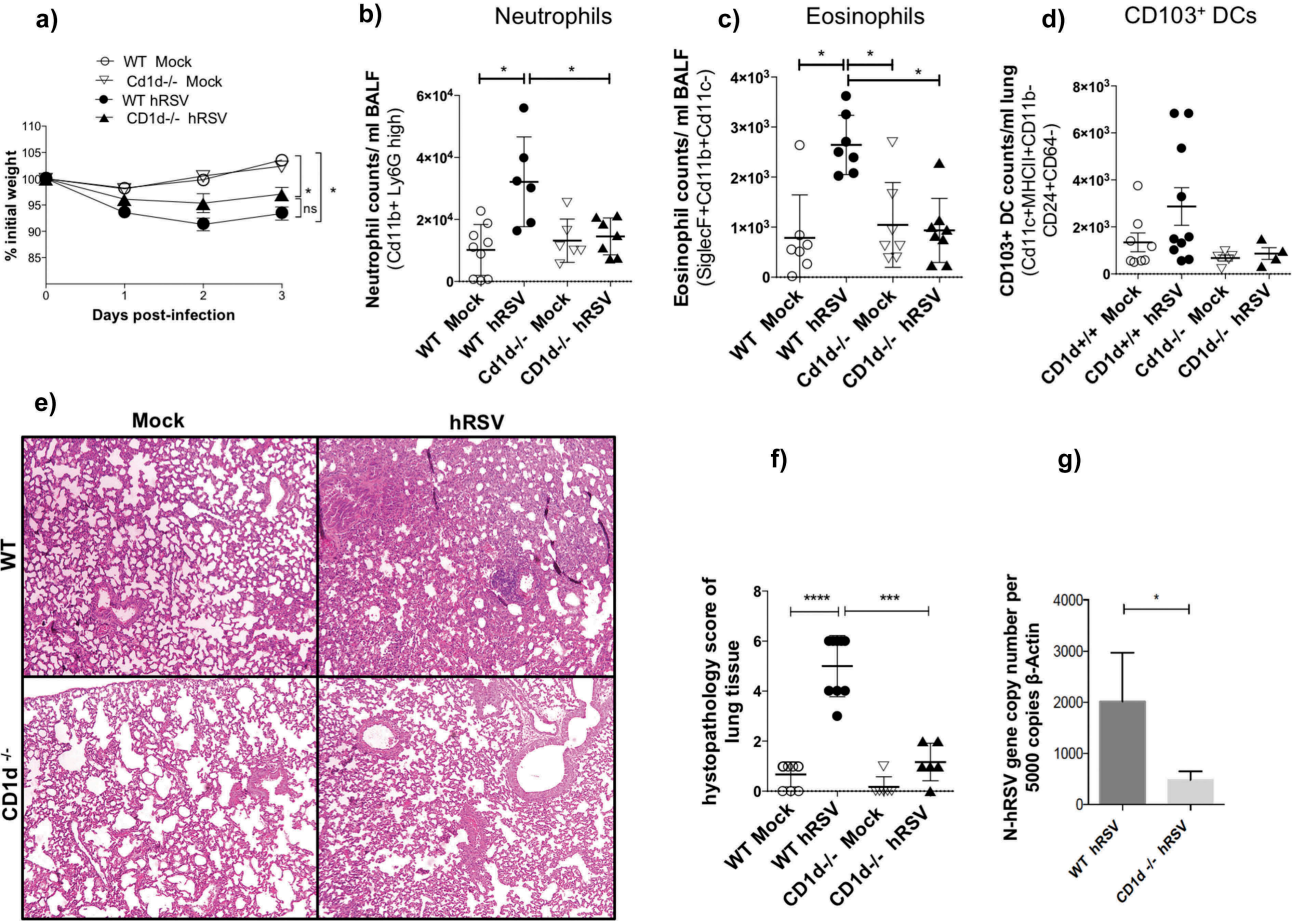
Infection with hRSV induces lower neutrophils, eosinophils, and CD103^+^ DCs infiltration into the lungs in CD1d-deficient mice. Wild-type [WT] and CD1d-deficient (CD1d^-/-^) BALB/c mice were infected with 1 × 10^6^ PFU of hRSV and 3 days after infection, BALF, and lungs were obtained. Bodyweight was measured for 3 days (a). Neutrophils (b), eosinophils (c), and CD103^+^DCs (d) in the BALF were evaluated by flow cytometry. Lung damage was evaluated by lung histology (e) and clinical score (f). Viral loads were evaluated by RT-qPCR (g). Combined data from three independent experiments (n = 2 to 3 mice per group and independent experiment) are shown. Kruskal-Wallis test and Mann–Whitney *U* test were performed to assess statistical differences. * P < 0.05. Bars represent mean ± SEM.

To evaluate the role of CD1d-restricted NKT cells during hRSV and hMPV infections, wild-type, and CD1d-deficient BALB/c (CD1d^-/-^) mice were infected with 1 × 10^6^ PFU of hRSV or hMPV. Three days post-infection, lungs were recovered for flow cytometry analyses and quantification of viral loads. The absence of the *cd1d* gene was previously corroborated in all CD1d-deficient mice by PCR and the lack of CD1d expression by flow cytometry (data not shown). As seen in Figure 2 and as previously reported [8,31], a loss of weight and high levels of infiltrating neutrophils and eosinophils to the lungs were observed in the hRSV-infected wild-type mice (Figure 2(a-c)). Furthermore, a tendency to higher numbers of infiltrating CD103^+^ DCs was found in hRSV-infected mice as compared to all the other groups evaluated (Figure 2(d)). Interestingly, we did not observe an increase in the total number of any of these cell types for hRSV-infected CD1d-deficient mice, displaying similar infiltrate values to the ones seen in the mock-treated mice (Figure 2(b-d)). This finding is consistent with the reduced degree of hRSV-associated disease found in hRSV-infected CD1d^−/-^mice, as compared to wild-type animals, as shown by H&E histological analyzes, and histopathological score (Figure 2(e,f)). Finally, we found reduced levels of viral loads in hRSV-infected CD1d^−/-^mice, as compared to wild-type mice (Figure 2(g)), altogether suggesting that the absence of CD1d, and therefore the lack of NKT cells, is reducing the severity of the hRSV-associated disease parameters.

**Figure 3.**
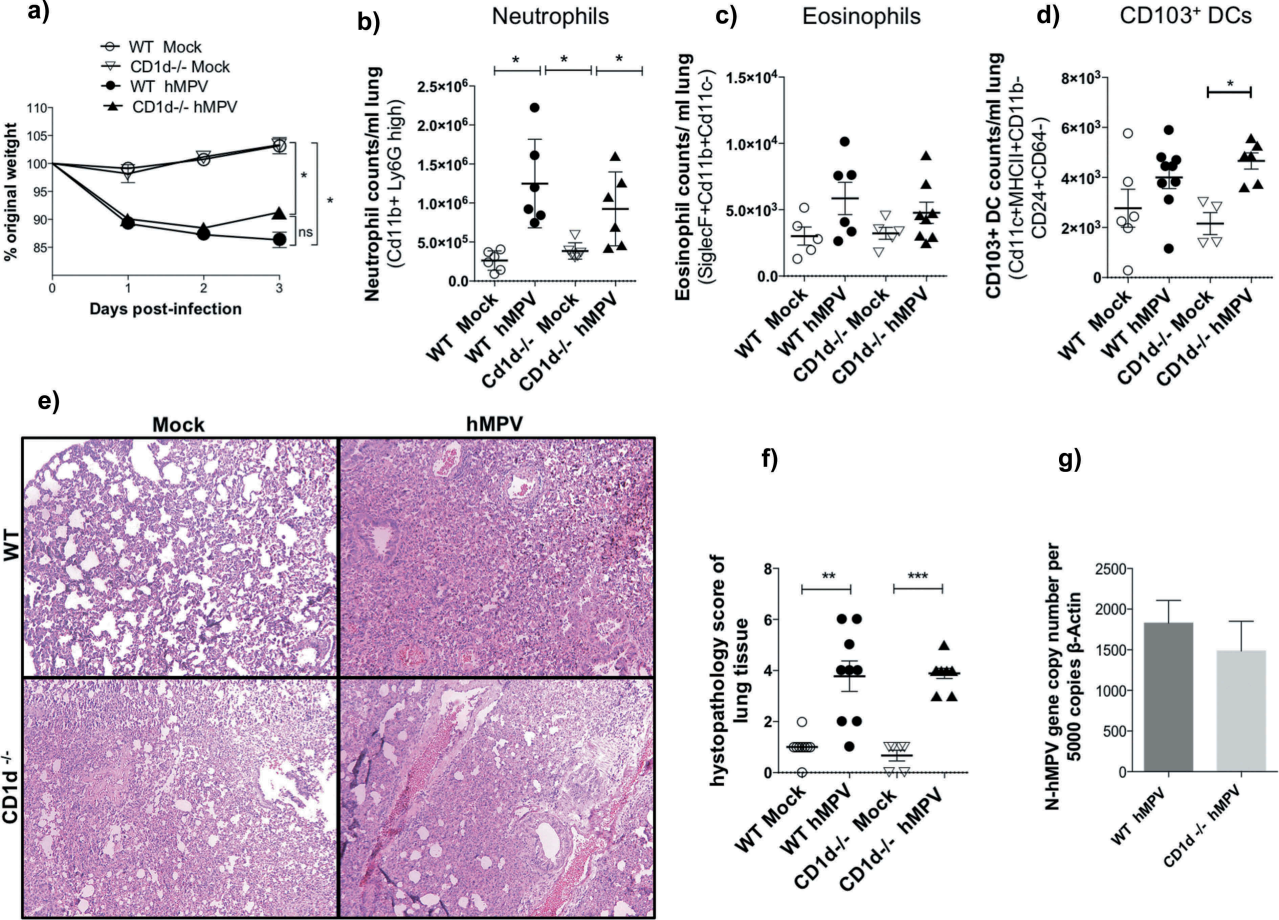
No significant changes were detected during an hMPV-infection between wild-type and CD1d-deficient BALB/c mice. Wild-type [WT] and CD1d-deficient BALB/c (CD1d^-/-^) mice were infected with 1 × 10^6^ PFU of hMPV and 3 days after infection BALF and lungs were obtained. Bodyweight was measured for 3 days (a). Neutrophils (b), eosinophils (c), and CD103^+^DCs (d) in the BALF were evaluated by flow cytometry. Lung damage was evaluated by lung histology (e) and clinical score (f). Viral loads were evaluated by RT-qPCR (g). Combined data from three independent experiments (n = 2 to 3 mice per group and independent experiment) are shown. Kruskal–Wallis test and Mann–Whitney *U* test were performed to assess statistical differences. * P < 0.05. Bars represent mean ± SEM.

When hMPV-infected animals were evaluated, we observed that this virus-induced an increase – or a tendency to increase – in the total number of infiltrating neutrophils, eosinophils and CD103^+^ DCs into the lungs, a marked loss of weight and a higher degree of lung damage, as compared to mock-treated mice (Figure 3(a-f)). However, in contrast to what was observed for hRSV, the phenotype seen for the hMPV-infected CD1d-deficient mice was similar for the levels of neutrophil, eosinophil, and CD103^+^ DCs infiltration, as compared to hMPV-infected wild-type control animals (Figure 3(b,d)). Further, histology analyzes and score of the lungs, along with the quantification of hMPV viral loads, showed an equivalent degree of lung damage and infection in both wild-type and CD1d-deficient mice (Figure 3(e,f)). These results suggest that the absence of NKT cells during an hMPV infection does not seems to play a beneficial role, contrary to what was seen for an hRSV infection, since no significant changes in the hMPV-associated diseases parameters where detected between the wild-type and the CD1d-deficient mice.

### NKT cells expanded after hRSV and hMPV infections show reduced cytokine secretion

**Figure 4.**
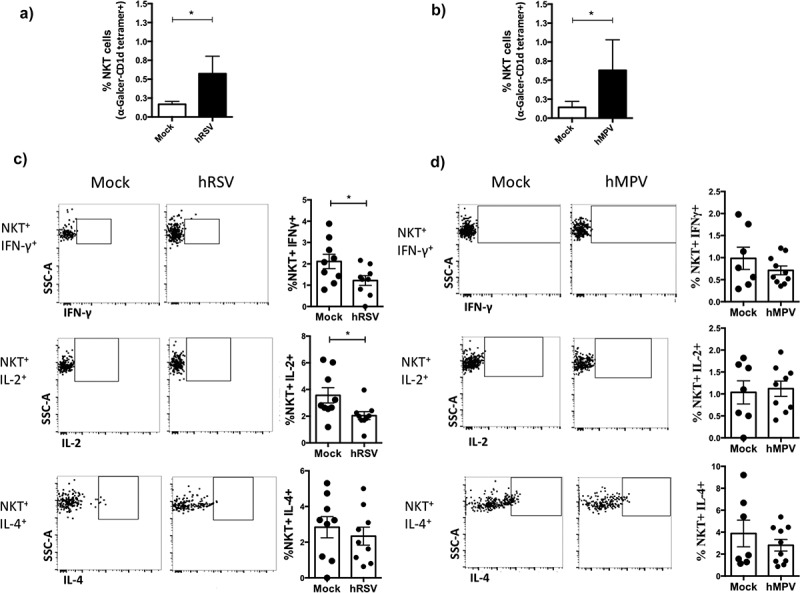
hRSV- and hMPV-mediated NKT activation induce lower cytokines levels than mock-treated mice in the lungs. Wild-type [WT] BALB/c mice were intranasally infected with 1 × 10^6^ PFU of hRSV or hMPV -or mock inoculated- and 3 days after infection, lungs were removed. Percentage of NKT cells in the lungs of hRSV (a) and hMPV (b) infected mice was assessed. Then, lungs cells were treated for the induction of the secretion of cytokines and intracellular staining of IFN-γ, IL-2, and IL-4 of NKT was performed (c) (d).Combined data from three independent experiments (n = 4–5 mice per group) are shown. Kruskal–Wallis test and Mann–Whitney *U* test were performed in order to assess statistical differences. * p < 0.05, ** p < 0.001. Bars represent mean ± SEM.

NKT cells are recruited into the lungs 3 days post-infection of hRSV and hMPV (Figure 4(a,b)). To evaluate whether the expanded NKT cells generated after hRSV and hMPV-infection display altered cytokine profiles, expression of IFN-γ, IL-2, and IL-4 were measured by flow cytometry in NKT derived from the lungs of infected wild-type BALB/c mice (Figure 4(c,d)). The observed high baseline production for some cytokines found in mock-treated-mice can be explained by the use of unspecific T cell stimulation with PMA and ionomycin. A decrease in the percentage of IFN-γ and IL-2 expressing NKT cells was found in the lungs from hRSV-infected mice as compared to mock-treated animals (Figure 4(c)). No statisticaldifferences were found in the frequency of the NKT^+^ IFN-γ^+^, NKT^+^ IL-4^+^, and NKT^+^ IL-2^+^from hMPV-infected as compared to mock-treated mice (Figure 4(d)). Thus, both viruses might be differently impairing NKT cells activation, as previously detected in the *in vitro* assays.

### Changes in the cytokine profiles in hRSV and hMPV-infected CD1d-deficient mice

**Figure 5.**
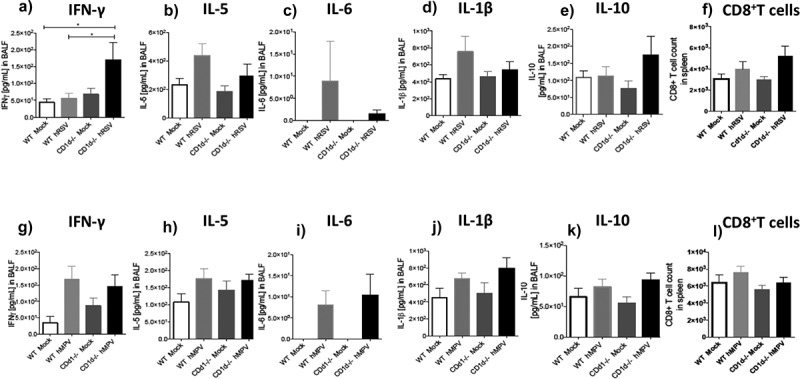
Cytokine profiling in BALF and CD8^+^ T cells population in mice infected with hRSV or hMPV. Wild-type [WT] and CD1d-deficient (CD1d^-/-^) BALB/c mice were infected with 1 × 10^6^ PFU of either hRSV or hMPV and 3 days after infection BALF was obtained. IFN-γ, IL-5, IL-6, IL-1β, and IL-10 were measured in those samples from hRSV- (a-e) and hMPV-infected (g-k) mice. CD8^+^ T cells population in spleen from hRSV- (f) and hMPV-infected (l) mice were measured. Combined data from three independent experiments (n = 2 to 3 mice per group and experiment) are shown. Kruskal–Wallis test and Mann–Whitney *U* test were performed in order to assess statistical differences. * P < 0.05. Bars represent mean ± SEM.

To characterize the cytokine profile in CD1d-deficient mice in response to either hRSV or hMPV infection, we evaluated the levels of IFN-γ, IL-5, IL-6, IL-1β, and IL-10 in BALFs 3 days after the viral challenge by ELISA assays (Figure 5). hRSV-infected CD1d-deficient mice showed increased levels of IFN-γ in BALF as compared to hRSV-infected wild-type mice (Figure 5(a)). In this line, a tendency to reduced levels of IL-5, IL-6, and IL-1β, in the BALF of hRSV-infected CD1d-deficient mice was detected, as compared to hRSV-infected wild-type mice (Figure 5(b-d)). A trend toward higher levels of IL-10 was also detected in the hRSV-infected CD1d^−/-^CD1d-deficient mice as compared to hRSV-infected wild-type mice (Figure 5(e)). Since we found higher levels of IFN-γ in the BALFs of CD1d-deficient mice as compared to wild-type mice, we evaluated the frequency of CD8^+^ T cells in response to hRSV infection, as these cells are mostly responsible for the secretion of said cytokine. Remarkably, the CD8^+^ T cell population tended to increase in the spleens of hRSV-infected CD1d-deficient mice, as compared to wild-type infected controls (Figure 5(f)). As previously seen, the increase of CD8^+^ T cells in wild-type infected mice was not marked, as compared to mock-treated mice. As for hMPV infection, CD1d-deficient mice exhibited similar levels of IFN-γ, IL-5, IL-6, IL-1β, and IL-10 in BALF as compared to wild-type mice (Figure 5(g-k)). Regarding the frequency of CD8^+^ T cell, similar frequencies were found among all the hMPV groups (Figure 5(l)). Thus, NKT cells may be modulating the secretion of IFN-γ, IL-5, IL-6, IL-1β, and IL-10 during hRSV infection, once again impairing the capacity of NKT cells to modulate the disease parameters.

## Discussion

Data from the present study suggest a differential contribution of NKT cells to the infection by either hRSV or hMPV in mice. Interestingly, CD1d-deficient BALB/c (CD1d^-/-^) mice presented a reduction of neutrophil, eosinophil, and CD103^+^ DCs infiltration, along with a reduction of lung damage and increased levels of IFN-γ. Moreover, we detected reduced levels of IL-5 secretion during hRSV infection, but not during hMPV infection. In this line, we describe for the first time the modulation of CD1d expression in alveolar macrophages, CD11b^+^ DCs, and epithelial cells in hRSV-infected mice, and alveolar macrophages and monocytes in hMPV-infected mice.

The role of NKT cells in both viral infections has been scarcely studied, as up to date only two reports related to hRSV and NKT cells are published, with partially controversial results [27,28]. In the first article published on these topics, it was shown that α-GalCer prevented the bodyweight loss associated with the hRSV disease and induced the secretion of high levels of IFN-γ, concluding that NKT cells play a protective role during hRSV infection [28]. Such alteration of hRSV-associated disease parameters in α-GalCer-treated mice were found related to an increase of hRSV specific-CD8^+^ T cells expansion. However, these authors also showed that viral loads were significantly higher in α-GalCer treated mice, as compared with vehicle-treated and hRSV-infected mice [27]. It is noteworthy that the time points characterized in Johnson *et al.* [28] study were close to 7 days, whereas our study was performed at 3 days post-infection with hRSV or hMPV; therefore there is a limitation in the possibility to contrast these studies. Contrary to this, it was recently found that neonatal NKT cell sensitization with α-GalCer exacerbates the pulmonary hRSV disease with an increment in the levels of IL-13, IL-4, and IL-5 in hRSV-reinfected adult mice [28]. Furthermore, after intraperitoneal injection of α-GalCer, the authors found a significant increase of infiltrating eosinophils in the lungs and similar amounts of neutrophils in the lungs, when compared with vehicle-treated and hRSV-infected mice. Also, these authors showed that CD1d-deficient mice had no significant weight loss after hRSV infection as compared to mock-treated animals [28]. Consistently with the latter publication, we found a reduction in the number of infiltrating neutrophils, eosinophils, and a trend towards a reduction of CD103^+^ DCs in the BALF of hRSV-infected CD1d^−/-^mice, indicating that NKT cells may be contributing to a detrimental immune response in hRSV infection. Importantly, these CD103^+^ DCs have been previously related to the priming of CD4^+^ and CD8^+^ T cells [39]. In this line, it has been previously reported that mice immunized with the FI-RSV vaccine -the first failed vaccine prototype for hRSV that resulted in the exacerbation of disease-related symptoms upon infection- also exhibited lower levels of CD103^+^ DCs. Therefore, we could suggest that this specific subset of DCs may be interacting with the iNKT cells population and, in this interaction, play a significant role in the modulation of the exacerbation of the disease caused by hRSV [39]. Consistent with such cell infiltration into the lungs, histopathological scores of lung tissue of hRSV-infected CD1d^−/-^ mice was significantly lower as compared to wild-type BALB/c mice. Therefore, hRSV could prompt the infiltration of inflammatory cells into the lungs –a classical sign of infection associated with this disease– by impairing NKT cell activation. Such an inhibition of NKT cells could be explained by a similar mechanism as the one previously described for conventional T cells [34], and inducing the secretion of Th-2 inflammatory cytokines.

As the infiltration of pro-inflammatory cells into the lungs plays a key role in the hRSV-associated disease [7], and differences in the levels of cell infiltration have been found in CD1d-deficient mice, the contribution of NKT cells might be relevant during an hRSV infection. The recruitment of immune cells into the lungs requires a specific cytokine environment [7], and in this line, in this work we observed statistically significant higher levels of IFN-γ, a tendency to lower levels of IL-5, IL-6, IL-1β, and increased levels of IL-10 in hRSV-infected CD1d-deficientmice, as compared to wild-type mice. Those cytokines are important in the hRSV-associated disease and the immune response to this virus. Accordingly, IL-5 is associated to an enhancement of RSV associated-pulmonary disease and reported as a predictor of asthma, following severe RSV bronchiolitis in children under 2 years old [40,41]. IL-1β is related to lung injury and inflammation [42], and herein the lower levels of IL-1β detected could explain the lower lung cell infiltration found in the CD1d-deficient hRSV-infected mice. Also, a correlation between lower levels of IL-10 in children and the severity of hRSV infection has been previously reported [43]. The higher levels of IFN-γ found in CD1d-deficient mice suggest that cells other than iNKT cells could be producing IFN-γ, the hallmark cytokine that will promote the clearance of the virus from the lung. It has been reported that influenza infection strongly promotes NKT cells-cytokine production, inducing an increase in the percentage of IFN-γ^+^ and IL-4^+^NKT cells, in mice [44]. However, the cytokine production by iNKT cells recruited to the lungs upon hMPV or hRSV infections does not seem to be that marked. This could be related to the different characteristics between influenza virus -Subphylum *Polyploviricotina*- and hRSV or hMPV –Subphylum *Haplaviricotina* [45]. Significantly, during an hRSV infection, the percentage of IFN-γ^+^and IL-2^+^ NKT^+^cells was significantly reduced in wild-type mice. However, such a reduction of cytokines secreting cells is low; therefore, it may not be relevant on the resolution of the infection or inflammation. Conversely, we detected similar levels of IFN-γ, IL-5, IL-6, IL-1β, and IL-10 in the BALF of hMPV-infected CD1d-deficient mice, as compared to hMPV-infected wild-type mice. These data are consistent with the levels of neutrophil infiltration detected in hMPV-infected CD1d-deficientmice, suggesting that NKT cells are not playing a significant role during hMPV-associated disease.

As previously reported, the T cell receptors in NKT cells recognize lipids or glycolipids presented by the CD1d molecule [18]. During viral infections, these lipids come from self-lipids expressed upon viral DNA or RNA recognition of antigen presenting cells and cytokine secretion [46]. It has been previously reported that viruses such as HSV-1[47], human cytomegalovirus [48], and HIV-1 modulate CD1d-mediated activation of NKT cells [22]. Such interference of CD1d has been previously reported in other viruses [22] but not for hRSV or hMPV. We found a modulation of CD1d in specific cell populations in both, hRSV- and hMPV-infected mice. Importantly, we found that CD1d expression was significantly modulated in alveolar macrophages, epithelial cells, and CD103^+^ DCs cells infiltrated into the lungs of hRSV-infected, as compared to uninfected mice. In contrast, a reduction of CD1d expression was detected in infiltrating alveolar macrophages and monocytes in hMPV-infected as compared to mock-treated mice. Such a difference in the modulation of CD1d in infiltrated cells into the lungs among these two viruses could explain the protection found in hRSV- but not in hMPV-infected CD1d-deficient mice. Importantly, the expression of CD1d is decreased in alveolar macrophages from hRSV– and hMPV– infected mice, and it has been previously reported that alveolar macrophages exert a protecting role in hRSV whereas being pathogenic during hMPV infection [49]. Therefore, modulation of CD1d expression in alveolar macrophages could explain the differences found between both viruses in CD1d-deficient mice. As described earlier, hRSV modulates the expression CD1d of some cells type, which along with the reduction of CD103^+^ DCs in the lungs from hRSV-infected CD1d^−/-^ mice, suggests that these cells might be inducing a potent proliferation of naïve CD8^+^ T cells through virus loading-derived peptides onto MHC-I molecules [50]. Thus, CD103^+^ DCs might be playing a role in lipid-mediated-CD1d activation of lymphoid cells in hRSV infection.

Importantly, it has been previously reported that the expression of CD1d in DCs and macrophages, but not in other cells such as B cells, mediates an iNKT Th_1_-like response [51]. Although similar CD1d expression levels were observed for mock-treated, hRSV-, and hMPV-infected DCs *in vitro*, significantly lower levels of IL-2 were found in hRSV- and hMPV-infected DCs when co-cultured with NKT cells. Thus, both hRSV and hMPV might impair iNKT cell function and cytokine production although this could be not related with CD1d levels expression. However, an impaired capacity to activate T cells was previously shown for DCs infected with either hRSV or hMPV thorough an inhibition of the proper assembly of the immunological synapse [13]. Such inhibition might also impair the capacity of hRSV- and hMPV-infected DCs to prime NKT cells, with the consequently reduced production of IL-2 in response to α-GalCer stimulation. Furthermore, it has been found that a protein of HSV-1 interferes with CD1d recycling from endosomal compartments, thereby inhibiting lipid antigen presentation by DCs [52]. Along this, although CD1d expression was not altered in all the infiltrated cells during either hRSV or hMPV infection, both viruses could be inhibiting the antigen presentation capacity of DCs and therefore ameliorating NKT cell activation and function. Consistently, it has been described that not only the down-regulation of CD1d cell surface expression can promote immune evasion by viruses, such as influenza and HSV-1, but also the modulation of the signal transduction pathways involved in the CD1d-restricted antigen presentation [52]. Interestingly, it has been described that two proteins of vaccinia virus can modulate CD1d antigen presentation [53]. Therefore, it is possible that both hRSV and hMPV could also display similar molecular mechanisms to ameliorate NKT cell function. Importantly, CD1d-deficient mice lack type I and type II NKT cells. Since type II NKT cells have been described to have more pronounced anti-inflammatory properties than NKT cells, it is possible that the effect we found in the mice lacking CD1d may be due to the unfullfiled role of both types of NKT cells during hRSV infection. According to the data mentioned above, the CD1d molecule seems to play a differential role in both hRSV and hMPV infections. Therefore, the effect of NKT cells and the modulation of CD1d are dependent on the infecting virus. Such a difference would suggest that hRSV could be promoting the secretion of cytokines that induced the activation of NKT cells. On the other hand, the lack of differences during an hMPV infection found between the wild-type and the CD1d-deficient mice could be associated with hMPV not inducing the activation or proliferation of the CD1d-restricted NKT cells. Due to the indicated importance of alveolar macrophages during hRSV and hMPV infections and the modulation of CD1d expression found in the lungs from hRSV- and hMPV-infected mice, further studies in NKT activation with alveolar macrophages are required and may provide more details about the mechanisms associated with the modulation of the disease by these viruses by NKT cells. Besides, the present work uncovers the detrimental effect of hRSV and hMPV on NKT cell function and could promote additional studies to elucidate the mechanism of impairment of NKT cell activity and the modulation of cytokine environment.

In conclusion, CD1d-restricted NKT cells seem to be detrimental during the disease caused by hRSV in mice as CD1d-deficientmice are less susceptible to hRSV, but not during an hMPV infection. Besides, hRSV can modulate the expression of CD1d in alveolar macrophages, CD11b^+^DCs, and epithelial cells, whereas hMPV only modulates such expression of CD1d on alveolar macrophages and monocytes. These data suggest a CD1d-dependent NKT cell activation for hRSV. Importantly, both hRSV and hMPV inhibit the activation of NKT cells as a mechanism for evading host immunity, displaying lower IL-2 production *in vitro*, a phenomenon that is not related to the capacity of these cells to prime a Th_1_ response, since no differences were detected in the secretion of IL-12.

## Supplementary Material

Supplemental Material
