# Supplementary material for: Contribution of NKT cells to the immune response and pathogenesis triggered by respiratory viruses
Source: Virulence. 2020 May 28;11(1):580–93. doi: 10.1080/21505594.2020.1770492 (PMC7549913; doi:10.1080/21505594.2020.1770492)
Supplement: Supplemental Material [file KVIR_A_1770492_SM9102.zip › Suppl captions.docx]

**Supplementary Figure 1. Profile of cytokine secretion by NKT cells hybridoma upon co-culture with pulsed and infected DCs*in vitro.*** Pre-pulsed α-GalCer and hRSV- (A, C, E) or hMPV- (B, D, F) infected DCs from wild-type C57BL/6 mice were co-cultured with iNKT cells hybridoma. The production of IL-12, IFN-γ, and IL-4 was evaluated by ELISA. UT: Untreated; VH: Vehicle; aGAL: α-Galcer. Combined data from three independent experiments (n=2-3 treatment per group and experiment) are shown. Kruskal-Wallis test was performed to assess statistical differences. Lines represent mean ± SEM.

**Supplementary Figure 2. Gating strategy to detect neutrophils, eosinophils and CD103^+^ DCs.** Lung and BALF from infected mice were stained and evaluated by flow cytometry. Dot plots of gating strategy and gated populations are shown as squares. After exclusion of doublets, CD45^+^ cells were selectedand the frequency of neutrophils (CD11c^-^CD11b^+^Ly6G^high^) (A), eosinophils (CD11c^-^CD11b^+^SiglecF^+^) (B),and CD103^+^ DCs (CD11c^+^ MHCII^+^CD11b^-^CD64^-^CD24^+^) (C),was evaluated.

**Supplementary Figure 3. Gating strategy to detect the expression of CD1d in infiltrated cells to the lung.**Infiltration into the lungs from virus-infected and mock-treated mice wasevaluated. Dot plots of gating strategy are shown. After exclusion of doublets, negative cells for CD45 marker were gated and then the positive cells for the Epcam markerspecific to epithelial cells were selected (CD45^-^Epcam^+^). CD45^+^ cells were gated to select CD103^+^ DCs (MHCII^high^CD11c^+^CD64^-^CD24^+^CD11b^-^), CD11b^+^DCs (MHCII^high^CD11c^+^CD64^-^CD24^+^CD11b^+^),alveolar macrophages (CD11b^-^CD11c^+^CD64^+^CD24^-^), monocytes (CD11b^+^MHCII^-^).After selecting cell populations, mean fluorescence intensity for CD1d positive population was evaluated using FlowJo v X 10.0.7 (FlowJo, LLC).

**Supplementary Figure 4. Gating strategy to detect the expression of intracellular cytokines produced by NKT cells from infected mice.** Intracellular staining for IL-2, IL-4 and IFN-γ cytokines were performed in the iNKT cells from the lungs of infected mice. A range between 2x10^5^ and 4x10^5^ events wasacquired byflow cytometer. After selection of the study population,doublets were excluded. On single cells, we selected the CD45^+^ cells and then over this population we selected the NKT cells with T cell receptor (TCR)β^+^α-Galcer-CD1d tetramer^+^. Finally, over the NKT cells gating, we selected the positive cells for each respective cytokine.
