# Supplementary figures and images for: Contribution of NKT cells to the immune response and pathogenesis triggered by respiratory viruses
Source: Virulence. 2020 May 28;11(1):580–93. doi: 10.1080/21505594.2020.1770492 (PMC7549913; doi:10.1080/21505594.2020.1770492)

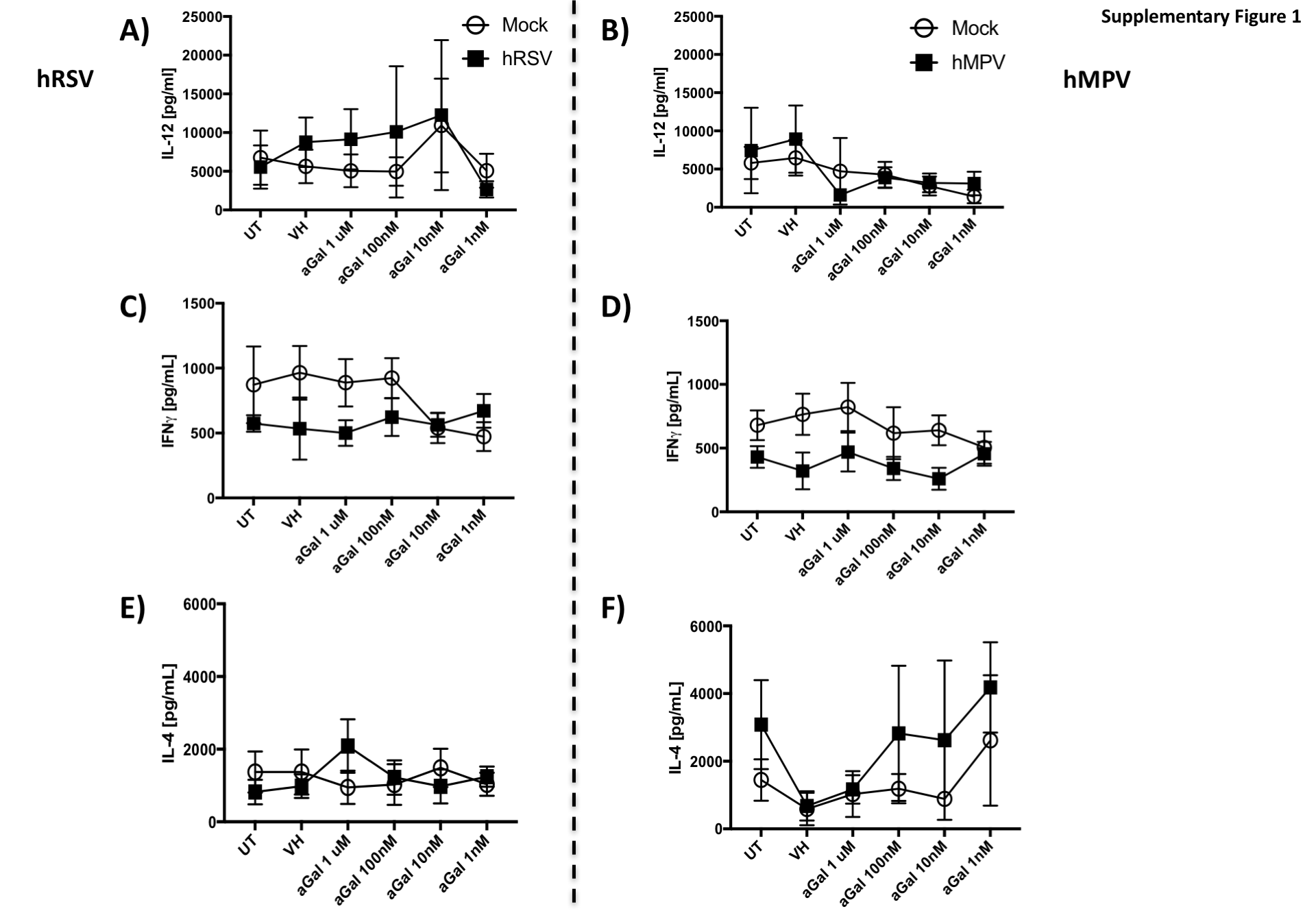

Supplement: Supplemental Material [file KVIR_A_1770492_SM9102.zip › Supp Fig 1.tiff]

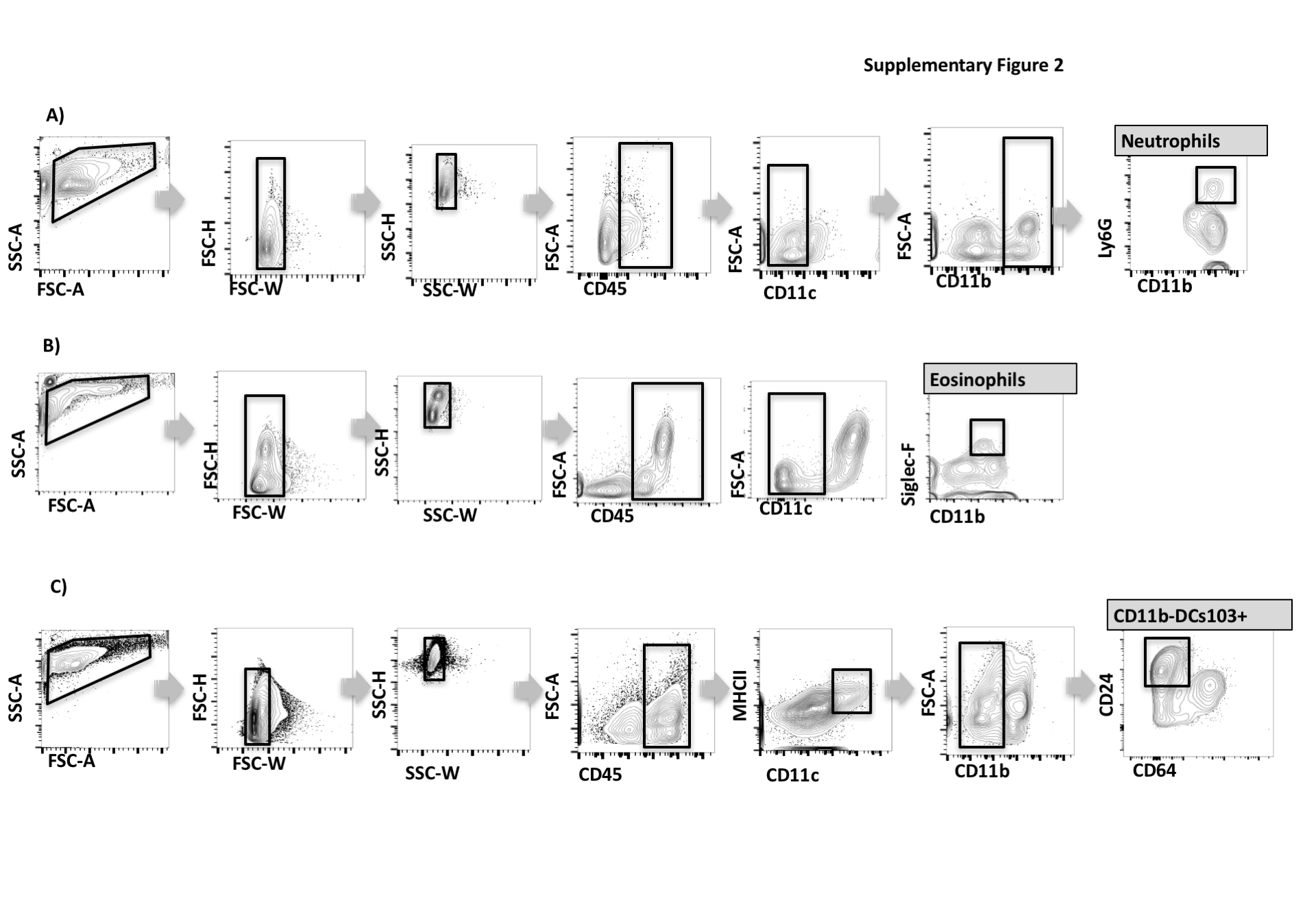

Supplement: Supplemental Material [file KVIR_A_1770492_SM9102.zip › Supp Fig 2.tiff]

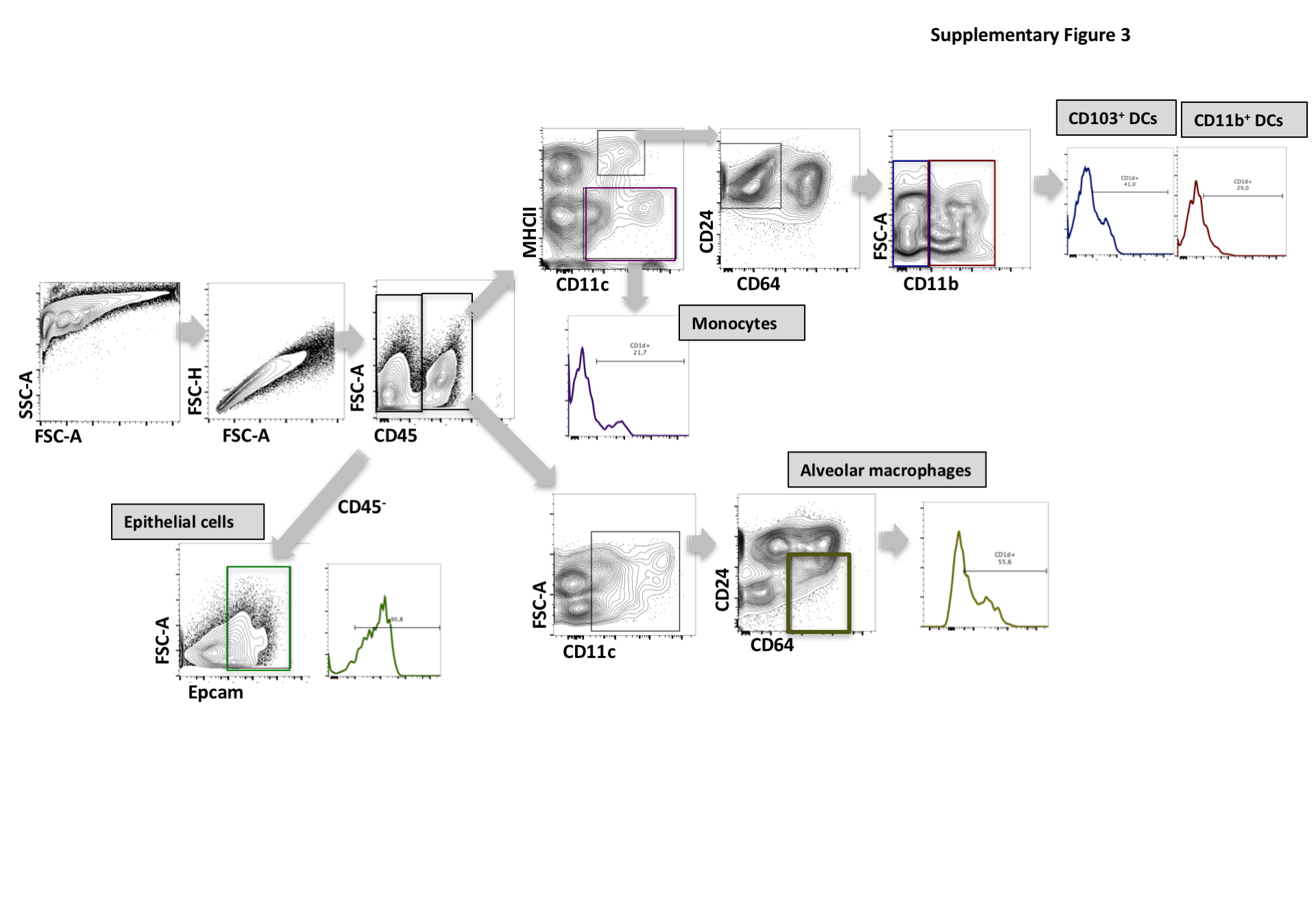

Supplement: Supplemental Material [file KVIR_A_1770492_SM9102.zip › Supp Fig 3.tiff]

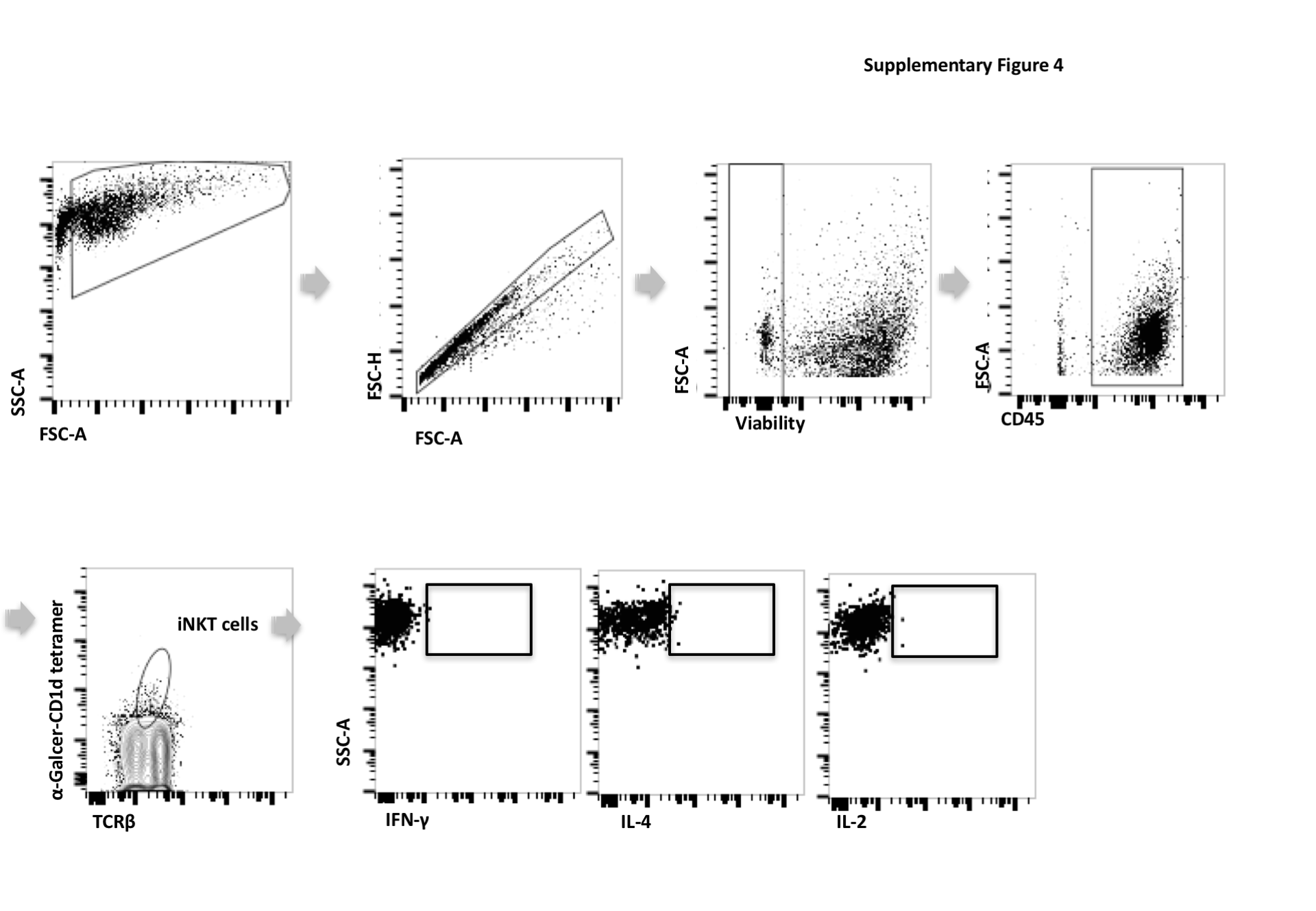

Supplement: Supplemental Material [file KVIR_A_1770492_SM9102.zip › Supp Fig 4.tiff]
